# Breakthrough SARS-CoV-2 infection in fully vaccinated patients with systemic lupus erythematosus: results from the COVID-19 Vaccination in Autoimmune Disease (COVAD) study

**DOI:** 10.1007/s00296-024-05682-6

**Published:** 2024-08-13

**Authors:** Leonardo Palazzo, Julius Lindblom, Emelie Kihlgren Olsson, Elena Nikiphorou, Chris Wincup, Sreoshy Saha, Syahrul Sazliyana Shaharir, Wanruchada Katchamart, Phonpen Akarawatcharangura Goo, Lisa Traboco, Yi-Ming Chen, James B. Lilleker, Arvind Nune, John D. Pauling, Vishwesh Agarwal, Dey Dzifa, Carlos Enrique Toro Gutiérrez, Carlo V. Caballero-Uribe, Hector Chinoy, COVAD Study Group, Bhupen Barman, Yogesh Preet Singh, Rajiv Ranjan, Avinash Jain, Sapan C. Pandya, Rakesh Kumar Pilania, Aman Sharma, Manesh Manoj M, Vikas Gupta, Chengappa G. Kavadichanda, Pradeepta Sekhar Patro, Sajal Ajmani, Sanat Phatak, Rudra Prosad Goswami, Abhra Chandra Chowdhury, Ashish Jacob Mathew, Padnamabha Shenoy, Ajay Asranna, Keerthi Talari Bommakanti, Anuj Shukla, Arun Kumar R. Pandey, Kunal Chandwar, Sinan Kardes, Döndü Üsküdar Cansu, Minchul Kim, Ashima Makol, Tulika Chatterjee, John D. Pauling, Chris Wincup, Lorenzo Cavagna, Nicoletta Del Papa, Gianluca Sambataro, Atzeni Fabiola, Marcello Govoni, Simone Parisi, Elena Bartoloni Bocci, Gian Domenico Sebastiani, Enrico Fusaro, Marco Sebastiani, Luca Quartuccio, Franco Franceschini, Pier Paolo Sainaghi, Giovanni Orsolini, Rossella De Angelis, Maria Giovanna Danielli, Vincenzo Venerito, Marcin Milchert, Lisa S. Traboco, Suryo Anggoro Kusumo Wibowo, Erick Adrian Zamora Tehozol, Jorge Rojas Serrano, Ignacio García-De La Torre, Jesús Loarce-Martos, Sergio Prieto-González, Albert Gil-Vila, Raquel Aranega Gonzalez, Masataka Kuwana, Akira Yoshida, Ran Nakashima, Shinji Sato, Naoki Kimura, Yuko Kaneko, Johannes Knitza, Stylianos Tomaras, Margarita Aleksandrovna Gromova, Or Aharonov, Tamer A. Gheita, Ihsane Hmamouchi, Leonardo Santos Hoff, Margherita Giannini, Francois Maurier, Julien Campagne, Alain Meyer, Melinda Nagy-Vincze, Daman Langguth, Vidya Limaye, Merrilee Needham, Nilesh Srivastav, Marie Hudson, Océane Landon-Cardinal, Syahrul Sazliyana Shaharir, Wilmer Gerardo Rojas Zuleta, José António Pereira Silva, João Eurico Fonseca, Olena Zimba, Vikas Agarwal, Latika Gupta, Ioannis Parodis

**Affiliations:** 1 Division of Rheumatology, Department of Medicine Solna, Karolinska Institutet and Karolinska University Hospital, Stockholm, Sweden; 2 Centre for Rheumatic Diseases, King’s College London, London, UK; 3 Rheumatology Department, King’s College Hospital, London, UK; 4 Division of Medicine, Department of Rheumatology, Rayne Institute, University College London, London, UK; 5 Centre for Adolescent Rheumatology Versus Arthritis at UCL, UCLH, GOSH, London, UK; 6 Mymensingh Medical College, Mymensingh, Bangladesh; 7 Faculty of Medicine, Universiti Kebangsaan Malaysia, Cheras, Kuala Lumpur, Malaysia; 8 Division of Rheumatology, Department of Medicine, Faculty of Medicine Siriraj Hospital, Mahidol University, Bangkok, Thailand; 9 Department of Medicine, Queen Savang Vadhana Memorial Hospital, Chonburi, Thailand; 10 Section of Rheumatology, Department of Medicine, St. Luke’s Medical Center-Global City, Taguig, Philippines; 11 Division of Allergy, Immunology and Rheumatology, Department of Internal Medicine, Taichung Veterans General Hospital, Taichung City, Taiwan, Republic of China; 12 Department of Medical Research, Taichung Veterans General Hospital, Taichung, Taiwan, Republic of China; 13 Division of Musculoskeletal and Dermatological Sciences, School of Biological Sciences, Faculty of Biology, Medicine and Health, Centre for Musculoskeletal Research, Manchester Academic Health Science Centre, The University of Manchester, Manchester, UK; 14 Manchester Centre for Clinical Neurosciences, Salford Royal NHS Foundation Trust, Salford, UK; 15 Southport and Ormskirk Hospital NHS Trust, Southport, UK; 16 Bristol Medical School Translational Health Sciences, Health Sciences, Bristol, UK; 17 Department of Rheumatology, North Bristol NHS Trust, Bristol, UK; 18 Mahatma Gandhi Mission Medical College, Navi Mumbai, Maharashtra India; 19 Rheumatology Unit, Department of Medicine and Therapeutics, University of Ghana Medical School, College of Health Sciences, Korle-Bu, Accra, Ghana; 20 Reference Center for Osteoporosis, Rheumatology and Dermatology, Pontificia Universidad Javeriana Cali, Cali, Colombia; 21 Department of Medicine, Hospital Universidad del Norte, Barranquilla, Atlantico Colombia; 22 National Institute for Health Research Manchester Biomedical Research Centre, Manchester University NHS Foundation Trust, The University of Manchester, Manchester, UK; 23 Department of Rheumatology, Salford Royal Hospital, Northern Care Alliance NHS Foundation Trust, Salford, UK; 24 Department of Clinical Immunology and Rheumatology, Sanjay Gandhi Postgraduate Institute of Medical Sciences, Lucknow, India; 25 Department of Rheumatology, Royal Wolverhampton Hospitals NHS Trust, Wolverhampton, UK; 26 Department of Rheumatology, Faculty of Medicine and Health, Örebro University, Örebro, Sweden

**Keywords:** COVID-19, Vaccination, Breakthrough infection, Systemic lupus erythematosus, Rheumatology, Autoimmune disease, Survey

## Abstract

**Objective:**

To determine the occurrence of breakthrough COVID-19 infections (BIs) in patients with systemic lupus erythematosus (SLE) compared with patients with other rheumatic autoimmune diseases (rAIDs), patients with non-rheumatic autoimmune diseases (nrAIDs), and healthy controls (HCs).

**Methods:**

The study was based on data from 7035 fully vaccinated respondents to the online COVAD questionnaire with SLE (N = 852), rAIDs (N = 3098), or nrAIDs (N = 414), and HCs (N = 2671). BI was defined as COVID-19 infection occurring in individuals vaccinated with ≥ 2 doses (or 1 dose of J&J) ≥ 14 days after vaccination and not after 6 months since the last vaccine dose. Data were analysed using linear and logistic regression models.

**Results:**

A total of 91/852 (10.7%) SLE patients reported at least one BI. The frequency of BIs in SLE patients was comparable to that among HCs (277/2671; *p* = 0.847) and patients with nrAID (39/414; *p* = 0.552) but higher than that among patients with other rAIDs (235/3098; *p* = 0.005). No demographic factors or treatments were associated with BIs in SLE patients (*p* ≥ 0.05 for all). Joint pain was more frequent in SLE patients than in HCs (odds ratio [OR]: 3.38; 95% confidence interval [CI]: 1.89–6.04; *p* < 0.001) or nrAID patients (OR: 2.44; 95% CI: 1.04–5.75; *p* = 0.041). Patient with SLE did not report a higher frequency of hospitalisation or need for advanced treatment for COVID-19 infection compared with disease controls and HCs, respectively.

**Conclusion:**

COVID-19 vaccination conferred similar protection against COVID-19 infection in terms of frequency and severity in patients with SLE to that reported by healthy individuals.

**Supplementary Information:**

The online version contains supplementary material available at 10.1007/s00296-024-05682-6.

## Introduction

The health consequences of coronavirus disease 2019 (COVID-19) infections have abated since the start of vaccination against the severe acute respiratory syndrome coronavirus-2 (SARS-CoV-2), which translates into less severe infections, decreased mortality rates, and lower hospitalisation rates [1–4]. A 10% increase in vaccination coverage has been reported to result in a 7% incidence reduction and 8% reduction in mortality from breakthrough infections (BIs) in the vaccinated healthy population [5]. The symptoms of COVID-19 infections have also been reported to be less severe in vaccinated compared with unvaccinated individuals [6].

Previous studies have suggested that in comparison with the general population, patients with rheumatic autoimmune diseases (rAIDs) such as systemic lupus erythematosus (SLE) are at higher risk of severe COVID-19 infection, yielding a hospitalisation frequency of 58% and mortality of 7% [7]. In patients with SLE, several factors may increase the vulnerability to severe COVID-19, including associated comorbidities and immunosuppressive treatments [8]. While glucocorticoids (GCs) are associated with a higher risk of hospitalisation due to COVID-19 infections [9, 10], the influence of other medications given for SLE, including conventional immunosuppressive drugs and biologics, still is controversial, and previous studies have reported conflicting results [9–12]. In addition, impaired immune responses in SLE patients, especially in those receiving immunosuppressive treatments, threatens vaccine effectiveness [13, 14].

As per current knowledge, recommendations suggest that the benefits from vaccination in people with rAIDs, including SLE, far outweigh the concerns related to ineffectiveness and vaccine-induced adverse events (AEs) [15, 16]. However, surveys on rAID patients’ attitudes to vaccination have reported vaccine hesitancy rates as high as 35%, with main concerns being uncertainty regarding vaccine safety and fear of vaccine-induced disease flares [17].

We recently showed that despite discrepancies across COVID-19 vaccines regarding a few minor AEs, those were overall well-tolerated by SLE patients irrespective of disease activity and background therapies [18]. However, there is still a dearth of data regarding COVID-19 BIs in patients with SLE, which framed the scope of the present investigation. Among questions that have yet to be addressed is the impact of disease activity or use of immunosuppressive or immunomodulatory therapies on the protection conferred from COVID-19 vaccines in patients with SLE. The aim of this study was to investigate the prevalence, characteristics, and predictors of COVID-19 BIs in patients with SLE compared with patients with rAIDs other than SLE, patients with non-rheumatic autoimmune diseases (nrAIDs), and healthy controls (HCs).

## Methods

### Study design and population

An international online, cross-sectional, multicentre survey-based study was conducted between January and May 2022 within the frame of the global COVID-19 Vaccination in Autoimmune Diseases (COVAD) collaboration. People over 18 years of age diagnosed with an autoimmune disease or considering themselves healthy answered questions regarding demographics, current medications, COVID-19 infections, and vaccination status. Some of the questions pertained to how many vaccine doses the participant had received and whether hospitalisation was required due to COVID-19 infection. The survey also included questions about symptoms during a COVID-19 infection, such as fever, cough, shortness of breath, loss of smell, loss of taste, running nose, and throat pain. In addition, participants were asked to report symptom duration for each symptom and whether advanced treatment was required for their COVID-19 infection [19, 20]. The survey did not impose any restriction to the respondents regarding the degree of disease activity or past or present organ involvement. The questionnaire was developed by international rheumatology experts based on literature review of relevant articles, and it was next pilot tested, validated, and translated into 18 languages. Details about the survey protocol and data collection are published elsewhere [19–21].

### Ethics

An initial segment in the online survey served to obtain informed consent from the study participants. The results are reported in compliance with the Checklist for Reporting Results of the Internet E-surveys (CHERRIES) [22]. The ethics committee of Sanjay Gandhi Postgraduate Institute of Medical Sciences (SGPGIMS) granted approval for the study (IEC code: 2021–143-IP-EXP-39).

### Clinical definitions

rAIDs listed in the questionnaire included SLE, ankylosing spondylitis, myositis and anti-synthetase syndrome, rheumatoid arthritis (RA), and systemic sclerosis (SSc), whereas nrAIDs included inflammatory bowel disease and type 1 diabetes, among others, as detailed elsewhere [20]. Survey respondents also had the option to specify their autoimmune disease in free text if not listed in the survey.

To define a BI, we adhered to the CDC’s definition, considering them to occur in vaccinated respondents i.e., those who have received either ≥ 2 doses or 1 dose of Johnson & Johnson (J&J; JNJ-78436735) ≥ 14 days after vaccination and not after 6 months since the last vaccine dose. Advanced treatment was considered as treatment with monoclonal antibodies.

### Statistical analysis

Descriptive data are presented as numbers (percentage) or means (standard deviation, [S.D.]), and medians (interquartile range, [IQR]) are indicated in case of non-normal distribution. The Pearson’s chi-squared (*χ*^2^) test was used to compare categorical variables. The duration of BIs in SLE patients and comparator groups was analysed by unadjusted and adjusted (for age, sex, and ethnicity) linear regression. Results from linear regression are presented as the coefficient (β), confidence interval (CI) and *p* value. Factors associated with BI among patients with SLE were investigated through univariable logistic regression. Differences in BI presentation between SLE patients and comparator groups were evaluated by multivariable logistic regression, adjusting for age, sex, and ethnicity. Results from logistic regression are presented as the odds ratio (OR), CI, and *p* value. For all analyses,* p* values < 0.05 were considered statistically significant. Analyses were performed and illustrations were created using the R Statistical Software version 4.3.3 (R Foundation for Statistical Computing, Vienna, Austria).

## Results

Out of 16,328 total respondents, we retrieved data from 10,783 survey respondents with complete responses. Of those, 7035 were fully vaccinated, including 852 (12.1%) patients with SLE, 3098 (44.0%) patients with rAID, 414 (5.9%) patients with nrAID, and 2671 HCs (38.0%), forming our study population (Supplementary Fig. S1**)**. Demographics and clinical features of SLE patients and comparator groups, including self-reported disease activity status, are presented in Table 1.

**Table 1:** Demographics and clinical data of the study population

	SLE (N = 852)	HC (N = 2671)	rAID (N = 3098)	nrAID (N = 414)
Patient characteristics				
Age; mean (S.D.)	41.2 (13.1); N = 839	39.9 (13.2); N = 2616	51.7 (14.1); N = 3051	44.7 (14.1); N = 402
Female gender; n (%)	798 (94.7); N = 843	1574 (59.8); N = 2631	2470 (80.7); N = 3062	339 (82.9); N = 409
Ethnicity; n (%)	N = 804	N = 2537	N = 2998	N = 397
African	80 (10.0)	88 (3.5)	127 (4.2)	3 (0.8)
Asian	255 (31.7)	693 (27.3)	591 (19.7)	56 (14.1)
Caucasian	270 (33.6)	739 (29.1)	1762 (58.8)	220 (55.4)
Hispanic	108 (13.4)	714 (28.1)	280 (9.3)	81 (20.4)
Others	91 (11.3)	303 (11.9)	238 (7.9)	37 (9.3)
Self-reported disease activity status; n (%)	N = 611	N = 34	N = 2500	N = 286
Inactive or in remission	187 (30.6)	4 (11.8)	473 (18.9)	84 (29.4)
Active but stable and manageable	258 (42.2)	19 (55.9)	1170 (46.8)	132 (46.2)
Active and improving	39 (6.4)	2 (5.9)	187 (7.5)	20 (7.0)
Active and worsening	70 (11.5)	5 (14.7)	422 (16.9)	17 (5.9)
Uncertain	57 (9.3)	4 (11.8)	248 (9.9)	33 (11.5)
Medications				
Biologics; n (%)				
Anti-TNF agents	4 (0.5)	2 (0.1)	318 (10.3)	9 (2.2)
Rituximab	25 (2.9)	0 (0.0)	207 (6.7)	2 (0.5)
Glucocorticoids; n (%)				
Glucocorticoid use	516 (60.6)	4 (0.1)	946 (30.5)	41 (9.9)
Prednisone eq. dose ≥ 10 mg/day	132 (15.5)	2 (0.1)	267 (8.6)	14 (3.4)
Hydroxychloroquine; n (%)	598 (70.2)	0 (0.0)	596 (19.2)	12 (2.9)
Immunosuppressants; n (%)				
Azathioprine	174 (20.4)	0 (0.0)	150 (4.8)	18 (4.3)
Calcineurin inhibitors	41 (4.8)	1 (0.0)	65 (2.1)	7 (1.7)
Cyclophosphamide	18 (2.1)	0 (0.0)	33 (1.1)	1 (0.2)
Leflunomide	20 (2.3)	0 (0.0)	173 (5.6)	0 (0.0)
Methotrexate	111 (13.0)	5 (0.2)	1077 (34.8)	13 (3.1)
Mycophenolic acid	166 (19.5)	1 (0.0)	256 (8.3)	4 (1.0)
Sulfasalazine	15 (1.8)	2 (0.1)	261 (8.4)	5 (1.2)
Intravenous immunoglobulin; n (%)	14 (1.6)	0 (0.0)	109 (3.5)	2 (0.5)
Small molecules; n (%)				
JAK inhibitors	2 (0.2)	0 (0.0)	81 (2.6)	1 (0.2)

Data are presented as numbers (percentage) or means (standard deviation). In case of missing values, numbers of patients with available data are indicated

*eq*. equivalent; *HC* healthy controls; *JAK* janus kinase; *nrAID* non-rheumatic autoimmune disease; *rAID* rheumatic autoimmune disease; *S.D*. standard deviation; *SLE* systemic lupus erythematosus; *TNF* tumour necrosis factor

**Fig. 1:**
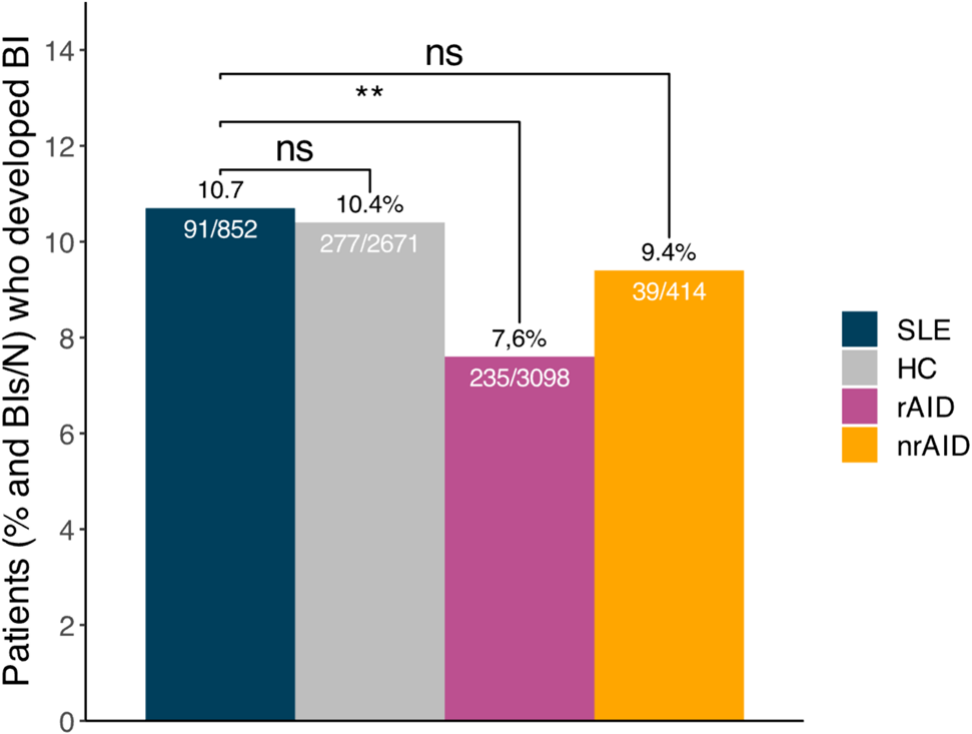
COVID-19 BIs in the study population. Bar plots depicting the proportion of patients with SLE, HC, patients with rAID, and patients with nrAID who reported a BI. *BI* breakthrough infection; *HC* healthy controls; *nrAID* non-rheumatic autoimmune disease; *rAID* rheumatic autoimmune disease; *SLE* systemic lupus erythematosus

Of the fully vaccinated SLE patients, 91/852 (10.7%) reported a BI. The frequency of BIs in fully vaccinated SLE patients was similar to that reported by HCs (277/2671; 10.4%; *p* = 0.847) and patients with nrAIDs (39/414; 9.4%; *p* = 0.552), but higher than that reported by patients with other rAIDs (235/3098; 7.6%; *p* = 0.005; Fig. 1).

### Characteristics of SLE patients reporting a BI

Among respondents with SLE who reported a BI, a vast majority was of female sex (N = 84; 93.3%), which was comparable to SLE patients not reporting a BI (N = 714; 94.8%). The mean (S.D.) age of SLE patients who reported a BI was 38.8 (11.1) compared to 41.5 (13.3) for patients not reporting a BI. Caucasians constituted the most represented ethnic group in SLE patients reporting a BI (N = 32; 36.8%), as well as in patients not reporting a BI (N = 238; 33.2%), followed by Asians (N = 29; 33.7% among SLE patients reporting a BI, and N = 226; 31.5% among SLE patients not reporting a BI).

**Fig. 2:**
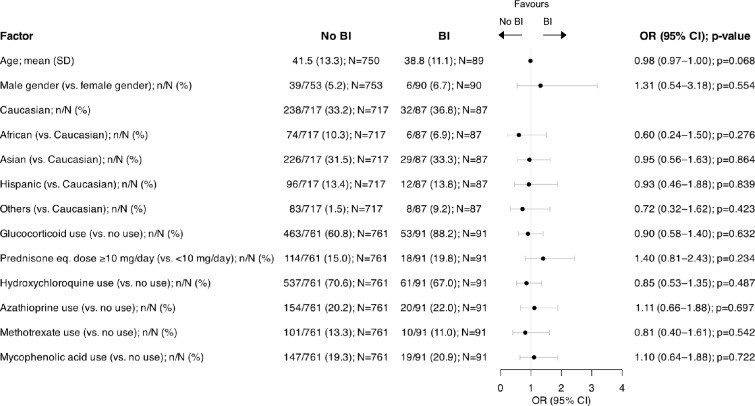
Factors associated with COVID-19 BIs in the SLE population. Forest plot illustrating results for SLE patients reporting a BI and SLE patients not reporting a BI, analysed by univariable logistic regression. *BI* breakthrough infection; *CI* confidence interval; *OR* odds ratio; *SLE* systemic lupus erythematosus

In univariable logistic regression analyses, we found no statistically significant association with the occurrence of BI among the analysed factors in our SLE population. Notably, GC use did not favour the occurrence of BI (OR: 0.90; 95% CI: 0.58–1.40; *p* = 0.632), nor did treatment with hydroxychloroquine (HCQ; OR: 0.85; 95% CI: 0.53–1.35; *p* = 0.487) or conventional immunosuppressive drugs, including azathioprine (AZA; OR: 1.11; 95% CI: 0.66–1.88; *p* = 0.697), methotrexate (MTX; OR: 0.81; 95% CI: 0.40–1.61; *p* = 0.542), or mycophenolic acid (MPA; OR: 1.10; 95% CI: 0.64–1.88;* p* = 0.722). Characteristics of SLE patients reporting a BI, compared with SLE patients not reporting a BI, are detailed in Fig. 2.

### Comparisons of BI characteristics between patients with SLE and HCs

**Fig. 3:**
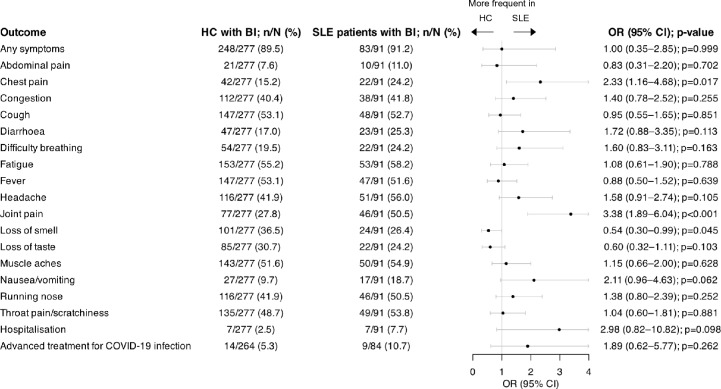
Characteristics of COVID-19 BIs in patients with SLE versus healthy controls. Forest plot illustrating results for SLE patients reporting a BI and HC reporting a BI, analysed by multivariable logistic regression adjusting for age, sex, and ethnicity. *BI* breakthrough infection; *CI* confidence interval; *HC* healthy controls; *OR* odds ratio; *SLE* systemic lupus erythematosus

The median (IQR) duration of BI was 10 [6–16] days in patients with SLE, and 8 [6–15] days in HCs, yielding no statistically significant difference in unadjusted (β = 0.98; 95% CI: -6.10–8.07; *p* = 0.786) and adjusted linear regression models (β = 0.12; 95% CI: -8.33–8.56; *p* = 0.978). In multivariable logistic regression adjusted for age, sex, and ethnicity, patients with SLE reported a higher occurrence of chest pain (OR: 2.33; 95% CI: 1.16–4.68; *p* = 0.017) and joint paint (OR: 3.38; 95% CI: 1.89–6.04; *p* < 0.001), but a lower occurrence of loss of taste (OR: 0.54; 95% CI: 0.30–0.99; *p* = 0.045) compared with HC. No other statistically significant differences in reported symptoms were found between the two groups. The proportion of SLE patients who reported a BI did not differ from the proportion of HCs who reported a BI with regard to hospitalisation (OR: 2.98; 95% CI: 0.82–10.82; *p* = 0.098) or the need for advanced treatment for COVID-19 (OR: 1.89; 95% CI: 0.62–5.77; *p* = 0.262). Characteristics of BIs in patients with SLE compared with HCs are detailed in Fig. 3**.**

### Comparisons of BI characteristics between patients with SLE and patients with other rAIDs

**Fig. 4:**
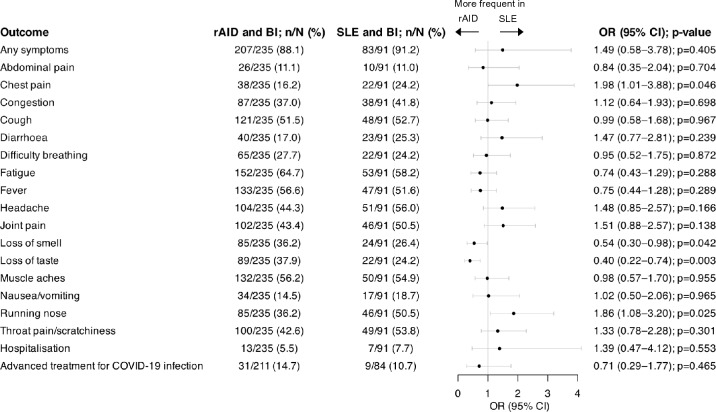
Characteristics of COVID-19 BIs in patients with SLE versus patients with rAID. Forest plot illustrating results for SLE patients reporting a BI and rAID patients reporting a BI, analysed by multivariable logistic regression adjusting for age, sex, and ethnicity. *BI* breakthrough infection; *CI* confidence interval; *OR* odds ratio; *rAID* rheumatic autoimmune disease; *SLE* systemic lupus erythematosus

The median (IQR) time to symptom resolution of BI was 14 [7–29] days in patients with rAIDs, which was longer compared to patients with SLE in unadjusted (β = − 14.00; 95% CI: − 25.42–2.59; *p* = 0.017) and adjusted (β = − 12.47; 95% CI: − 24.80–0.14; *p* = 0.048) linear regression models. The overall occurrence of any symptoms was comparable between the two groups (OR: 1.49; 95% CI: 0.58–3.78; *p* = 0.405). Compared with patients with other rAIDs, patients with SLE more frequently experienced chest pain (OR: 1.98; 95% CI: 1.01–3.88; *p* = 0.046) and running nose (OR: 1.86; 95% CI: 1.08–3.20; *p* = 0.025), and less frequently experienced loss of smell (OR: 0.54; 95% CI: 0.30–0.98; *p* = 0.042) and loss of taste (OR: 0.40; 95% CI: 0.22–0.74; *p* = 0.003). No significant difference was found in hospitalisation frequencies (OR: 1.39; 95% CI: 0.47–4.12; *p* = 0.553) or the need for advanced treatment for COVID-19 (OR: 0.71; 95% CI: 0.25–1.77; *p* = 0.465) between the two groups. Results from multivariable logistic regression of factors associated with BI between patients with SLE and patients with other rAIDs are illustrated in Fig. 4.

### Comparisons of BI characteristics between patients with SLE and patients with nrAIDs

**Fig. 5:**
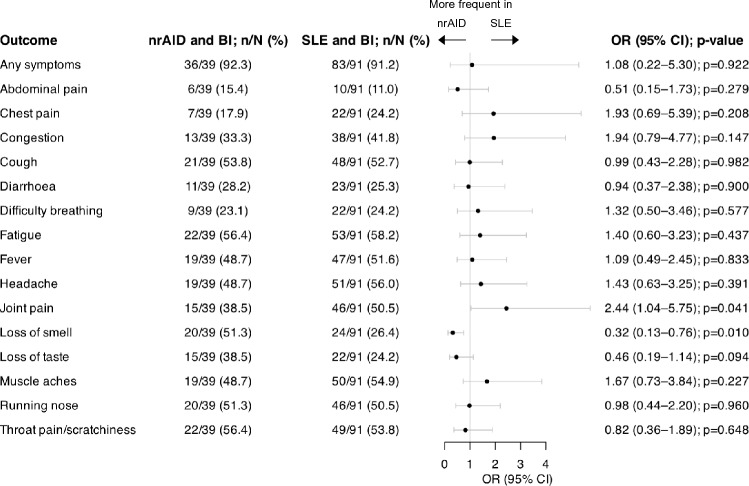
Characteristics of COVID-19 BIs in patients with SLE versus patients with nrAID. Forest plot illustrating results for SLE patients reporting a BI and nrAID patients reporting a BI, analysed by multivariable logistic regression adjusting for age, sex, and ethnicity. *BI* breakthrough infection; *CI* confidence interval; *nrAID* non-rheumatic autoimmune disease; *OR* odds ratio; SLE systemic lupus erythematosus

The median (IQR) BI duration was 10 (4–22.5) days in patients with nrAID and did not differ from that in patients with SLE in unadjusted (β = − 0.81; 95% CI: − 8.53–6.92; *p* = 0.838) and adjusted (β = − 0.25; 95% CI: − 8.78–8.28; *p* = 0.954) linear regression models. In multivariable logistic regression analysis, the overall frequency of any reported symptoms was similar between the two groups (OR: 1.08; 95% CI: 0.22–5.30; *p* = 0.922). Patients with SLE reported higher occurrence of joint pain (OR: 2.44; 95% CI: 1.04–2.75; *p* = 0.041), but lower occurrence of loss of smell (OR: 0.32; 95% CI: 0.13–0.76; *p* = 0.010). No other BI characteristics were found to differ between the two groups. Results are shown in Fig. 5.

## Discussion

Despite the multitude of studies on COVID-19 infection and COVID-19 vaccines since the start of the pandemic, there still exists a dearth of generalisable data on BIs in patients with SLE. In the present study, we investigated COVID-19 BIs in patients with SLE compared with patients with rAIDs other than SLE, patients with nrAIDs, and HCs using data from the COVAD study. Our findings indicate that fully vaccinated SLE patients experience BIs with similar frequency compared to fully vaccinated HCs. In addition, the severity of reported BIs was comparable between SLE patients and HCs, and between SLE patients and disease controls, as were hospitalisation frequencies and the need for advanced treatment for COVID-19 infection.

We found the self-reported frequency of BIs to be 10.7% in SLE patients and 10.4% in HCs, which is higher than the reported rates in other cohorts of patients with rAIDs and nrAIDs, using the same definition of BI [23–25]. One survey from Southern India reported a prevalence of BIs of 1.1% among patients with rAIDs [23], and data from the EULAR COVAX registry demonstrated a frequency of BI of 0.7% in patients with rAIDs, and 1.1% in patients with nrAID [24]. Another study from the US found that in patients with rAIDs who developed a COVID-19 infection, only 4.7% of the infections were BIs [25]. These differences might be due to our study design, which was susceptible to selection and recall bias, meaning that patients who were more willing to respond to the e-survey were probably more likely to have had a COVID-19 infection.

In this study, we found that the reported frequency and severity of BIs was comparable in patients with SLE and HCs. Therefore, SLE per se does not seem to predispose to BI in fully vaccinated subjects, nor is it a risk factor for the degree of severity of a BI or poorer outcome post infections. Patients with rAIDs constitute a vulnerable population with a higher incidence of infections, including COVID-19 infection, and previous studies suggested that SLE patients were at an increased risk of hospitalisation due to COVID-19 infection [26–28]. However, whether SLE patients are at an increased risk of severe COVID-19 BIs remains controversial. Although some reports have highlighted an elevated risk of hospitalisation in this patient population when experiencing a COVID-19 BI [29], other studies have reported no difference in incidence or severe complications of COVID-19 BI between vaccinated SLE patients and the vaccinated general population [30]. Notably, in a previous study from the UK exploring the risk of COVID-19 among unvaccinated and vaccinated patients with SLE, defined as having at least one dose of a COVID-19 vaccine, the authors found a higher risk of COVID-19 infection and severe sequelae in unvaccinated SLE patients compared with unvaccinated HCs, but no differences among the vaccinated population [30]. Our finding of non-significant difference in BIs prevalence between patients with SLE and HCs is in the same direction, offering evidence that COVID-19 vaccination confers adequate protection to this high-risk population.

We observed no major differences between patients with SLE and patients with other rAIDs with regard to COVID-19 BI symptoms. However, patients with rAIDs were less likely to develop a BI compared with patients with SLE, which may be indicative of the vulnerability of the SLE population to viral infections. This might also be due to immunosuppressive drugs commonly used in SLE, e.g., mycophenolate mofetil which has been shown to impede immune responses to vaccination [31]. In addition, our finding may be explained by heightened awareness and increased perception of COVID-19 infection consequences in this control group, which included individuals with high coexistent cardiopulmonary disease burden, such as patients with SSc or anti-synthetase syndrome [32–34]. This may have led to protective behaviours taken by this population, like physical distancing and shielding [35].

In our cohort of SLE patients, we did not identify any predictors of BI among the analysed clinical and demographical factors. We observed that the use of immunomodulating drugs, including HCQ, MPA, AZA, and MTX, was not associated with an increased odds of experiencing a BI. The use of immunosuppressants is a known risk factor for developing a COVID-19 BI [36, 37]. However, a study from the Netherlands reported that immunosuppressive therapy among patients with immune-mediated inflammatory disease was not associated with a higher incidence of BI in non-immunocompromised patients and HCs [38]. Previous studies have showed that the use of immunosuppressive agents, such as high daily doses of prednisone and belimumab, may be associated with a weak antibody response to COVID-19 vaccines [39, 40]. In the present study, we could not explore the relationship between biologics, including B cell targeting therapies, and the incidence of BIs due to the low number of SLE patients treated with these drugs among the respondents. Thus, further investigation is warranted for the exploration of these associations.

In our study, the overall frequency of any symptoms was similar among patients with SLE compared with disease groups. The increased occurrence of joint pain during BI in patients with SLE compared to HCs and with patients with nrAIDs, but not in patients with other rAIDs, may be due to underlying osteoarticular involvement, which is frequent in patients with rAIDs. COVID-19 has been associated with rheumatic musculoskeletal manifestations [41], and there is evidence that COVID-19 and rAIDs share some pro-inflammatory pathways [42]. Thus, our findings suggest that COVID-19 may act as a trigger for exacerbating symptoms in patients with chronically inflamed joints in the context of an underlying pre-existing rheumatic disease, including SLE.

One important limitation of this study was that it relied on an online survey, which introduces selection bias. Hence, individuals without internet access and those from lower socioeconomic regions are likely to be less represented in the sampling [43]. Secondly, being based on a self-reported questionnaire, our study lacked data on mortality. Moreover, clinical manifestations as per standard practice in SLE studies, e.g., using tools such as Systemic Lupus Erythematosus Disease Activity Index 2000 (SLEDAI) [44] or British Isles Lupus Assessment Group (BILAG) [45], were not captured. Lastly, the self-reporting nature of the questionnaire introduces recall bias. Nonetheless, a major strength was that COVAD is one of the largest studies of COVID-19, encompassing a global sample that is diverse with respect to demographics and comorbidities. Furthermore, the questionnaire included an extensive list of factors, including patient individual demographics, number of vaccine doses, therapy received, and details on BI clinical presentation, which facilitated in-depth analysis of multiple factors in relation to COVID-19 BIs.

In summary, we herein report a comparable prevalence of COVID-19 BIs across fully vaccinated SLE patients, HCs, and patients with nrAIDs, though a higher prevalence compared to that of patients with rAIDs. Our results suggest that COVID-19 vaccination confers similar protection against COVID-19 infection, in terms of frequency and severity, in patients with SLE compared with the healthy population. Finally, along with previous studies which have been reassuring concerning vaccine safety [18], our findings lend support for encouraging full vaccination in patients with SLE.

## Conflict of interest

E.N. has received speaker honoraria/participated in advisory boards for Celltrion, Pfizer, Sanofi, Gilead, Galapagos, AbbVie, and Lilly, and holds research grants from Pfizer and Lilly. J.B.L. has received speaker honoraria/participated in advisory boards for Sanofi Genzyme, Roche, and Biogen. None is related to this manuscript. H.C. was supported by the National Institution for Health Research Manchester Biomedical Research Centre Funding Scheme. The views expressed in this publication are those of the authors and not necessarily those of the NHS, National Institute for Health Research, or Department of Health. H.C. has received grant support from Eli Lilly and UCB, consulting fees from Novartis, Eli Lilly, Orphazyme, Astra Zeneca, speaker for UCB, and Biogen. I.P. has received research funding and/or honoraria from Amgen, AstraZeneca, Aurinia Pharmaceuticals, Elli Lilly and Company, Gilead Sciences, GlaxoSmithKline, Janssen Pharmaceuticals, Novartis and F. Hoffmann-La Roche AG. The other authors have no conflict of interest relevant to this manuscript.

## Supplementary Information

Below is the link to the electronic supplementary material.

Supplementary file1 (DOCX 66 KB)

Supplementary file2 (DOCX 36 KB)

## Data Availability

The datasets generated and/or analyzed during the current study are not publicly available but are available from the corresponding author upon reasonable request.

## References

[1] Haas EJ, Angulo FJ, McLaughlin JM, Anis E, Singer SR, Khan F et al (2021) Impact and effectiveness of mRNA BNT162b2 vaccine against SARS-CoV-2 infections and COVID-19 cases, hospitalisations, and deaths following a nationwide vaccination campaign in Israel: an observational study using national surveillance data. Lancet 397(10287):1819–1829 33964222 10.1016/S0140-6736(21)00947-8 PMC8099315

[2] Lopez Bernal J, Andrews N, Gower C, Robertson C, Stowe J, Tessier E et al (2021) Effectiveness of the Pfizer-BioNTech and Oxford-AstraZeneca vaccines on covid-19 related symptoms, hospital admissions, and mortality in older adults in England: test negative case-control study. BMJ 373:n1088 33985964 10.1136/bmj.n1088 PMC8116636

[3] Watson OJ, Barnsley G, Toor J, Hogan AB, Winskill P, Ghani AC (2022) Global impact of the first year of COVID-19 vaccination: a mathematical modelling study. Lancet Infect Dis 22(9):1293–1302 35753318 10.1016/S1473-3099(22)00320-6 PMC9225255

[4] Liu Q, Qin C, Liu M, Liu J (2021) Effectiveness and safety of SARS-CoV-2 vaccine in real-world studies: a systematic review and meta-analysis. Infect Dis Poverty 10(1):132 34776011 10.1186/s40249-021-00915-3 PMC8590867

[5] Suthar AB, Wang J, Seffren V, Wiegand RE, Griffing S, Zell E (2022) Public health impact of covid-19 vaccines in the US: observational study. BMJ 377:e069317 35477670 10.1136/bmj-2021-069317 PMC9044401

[6] Lee CJ, Woo W, Kim AY, Yon DK, Lee SW, Koyanagi A et al (2022) Clinical manifestations of COVID-19 breakthrough infections: a systematic review and meta-analysis. J Med Virol 94(9):4234–4245 35588301 10.1002/jmv.27871 PMC9348075

[7] Xu C, Yi Z, Cai R, Chen R, Thong BY, Mu R (2021) Clinical outcomes of COVID-19 in patients with rheumatic diseases: a systematic review and meta-analysis of global data. Autoimmun Rev 20(4):102778 33609804 10.1016/j.autrev.2021.102778 PMC7889462

[8] Mehta P, Gasparyan AY, Zimba O, Kitas GD (2022) Systemic lupus erythematosus in the light of the COVID-19 pandemic: infection, vaccination, and impact on disease management. Clin Rheumatol 41(9):2893–2910 35639259 10.1007/s10067-022-06227-7 PMC9152659

[9] Ugarte-Gil MF, Alarcón GS, Izadi Z, Duarte-García A, Reátegui-Sokolova C, Clarke AE et al (2022) Characteristics associated with poor COVID-19 outcomes in individuals with systemic lupus erythematosus: data from the COVID-19 Global Rheumatology Alliance. Ann Rheum Dis 81(7):970–978 35172961 10.1136/annrheumdis-2021-221636 PMC8882632

[10] Gendebien Z, von Frenckell C, Ribbens C, André B, Thys M, Gangolf M et al (2021) Systematic analysis of COVID-19 infection and symptoms in a systemic lupus erythematosus population: correlation with disease characteristics, hydroxychloroquine use and immunosuppressive treatments. Ann Rheum Dis England 80:e94 10.1136/annrheumdis-2020-218244 32586921

[11] Ramirez GA, Argolini LM, Bellocchi C, Moroni L, Della-Torre E, Farina N et al (2021) Impact of the COVID-19 pandemic in patients with systemic lupus erythematosus throughout one year. Clin Immunol 231:108845 34478882 10.1016/j.clim.2021.108845 PMC8405598

[12] Marques CDL, Kakehasi AM, Pinheiro MM, Mota LMH, Albuquerque CP, Silva CR et al (2021) High levels of immunosuppression are related to unfavourable outcomes in hospitalised patients with rheumatic diseases and COVID-19: first results of ReumaCoV Brasil registry. RMD Open 7(1):e001461corr1 33510041 10.1136/rmdopen-2020-001461 PMC7844930

[13] Sarin KY, Zheng H, Chaichian Y, Arunachalam PS, Swaminathan G, Eschholz A et al (2024) Impaired innate and adaptive immune responses to BNT162b2 SARS-CoV-2 vaccination in systemic lupus erythematosus. JCI Insight. 10.1172/jci.insight.176556 38456511 10.1172/jci.insight.176556 PMC10972586

[14] Ferri C, Ursini F, Gragnani L, Raimondo V, Giuggioli D, Foti R et al (2021) Impaired immunogenicity to COVID-19 vaccines in autoimmune systemic diseases High prevalence of non-response in different patients’ subgroups. J Autoimmun 125:102744 34781162 10.1016/j.jaut.2021.102744 PMC8577991

[15] Curtis JR, Johnson SR, Anthony DD, Arasaratnam RJ, Baden LR, Bass AR et al (2023) American college of rheumatology guidance for COVID-19 vaccination in patients with rheumatic and musculoskeletal diseases: version 5. Arthritis Rheumatol 75(1):E1–E16 36345691 10.1002/art.42372 PMC9878068

[16] Landewé RBM, Kroon FPB, Alunno A, Najm A, Bijlsma JW, Burmester GR et al (2022) EULAR recommendations for the management and vaccination of people with rheumatic and musculoskeletal diseases in the context of SARS-CoV-2: the November 2021 update. Ann Rheum Dis 81(12):1628–1639 35197264 10.1136/annrheumdis-2021-222006

[17] Ko T, Dendle C, Woolley I, Morand E, Antony A (2021) SARS-COV-2 vaccine acceptance in patients with rheumatic diseases: a cross-sectional study. Hum Vaccin Immunother 17(11):4048–4056 34357827 10.1080/21645515.2021.1958611 PMC8828082

[18] Naveen R, Nikiphorou E, Joshi M, Sen P, Lindblom J, Agarwal V et al (2023) Safety and tolerance of vaccines against SARS-CoV-2 infection in systemic lupus erythematosus: results from the COVAD study. Rheumatology (Oxford) 62(7):2453–2463 36413073 10.1093/rheumatology/keac661 PMC10321116

[19] Sen P, Gupta L, Lilleker JB, Aggarwal V, Kardes S, Milchert M et al (2022) COVID-19 vaccination in autoimmune disease (COVAD) survey protocol. Rheumatol Int 42(1):23–29 34779868 10.1007/s00296-021-05046-4 PMC8591970

[20] Fazal ZZ, Sen P, Joshi M, Ravichandran N, Lilleker JB, Agarwal V et al (2022) COVAD survey 2 long-term outcomes: unmet need and protocol. Rheumatol Int 42(12):2151–2158 35964271 10.1007/s00296-022-05157-6 PMC9376047

[21] Gaur PS, Zimba O, Agarwal V, Gupta L (2020) Reporting survey based studies - a primer for authors. J Korean Med Sci 35(45):e398 33230988 10.3346/jkms.2020.35.e398 PMC7683244

[22] Eysenbach G (2004) Improving the quality of Web surveys: the Checklist for Reporting Results of Internet E-Surveys (CHERRIES). J Med Internet Res. Canada 6:e34 10.2196/jmir.6.3.e34 PMC1550605 15471760

[23] Mohanasundaram K, Santhanam S, Natarajan R, Murugesan H, Nambi T, Chilikuri B et al (2022) Covid-19 vaccination in autoimmune rheumatic diseases: a multi-center survey from southern India. Int J Rheum Dis 25(9):1046–1052 35773944 10.1111/1756-185X.14378 PMC9349850

[24] Machado PM, Lawson-Tovey S, Strangfeld A, Mateus EF, Hyrich KL, Gossec L et al (2022) Safety of vaccination against SARS-CoV-2 in people with rheumatic and musculoskeletal diseases: results from the EULAR Coronavirus Vaccine (COVAX) physician-reported registry. Ann Rheum Dis 81(5):695–709 34972811 10.1136/annrheumdis-2021-221490

[25] Cook C, Patel NJ, D’Silva KM, Hsu TY, DiIorio M, Prisco L et al (2022) Clinical characteristics and outcomes of COVID-19 breakthrough infections among vaccinated patients with systemic autoimmune rheumatic diseases. Ann Rheum Dis. England 81:289–91 10.1136/annrheumdis-2021-221326 PMC8791428 34489304

[26] Cordtz R, Kristensen S, Dalgaard LPH, Westermann R, Duch K, Lindhardsen J et al (2021) Incidence of COVID-19 hospitalisation in patients with systemic lupus erythematosus: a Nationwide Cohort Study from Denmark. J Clin Med 10(17):3842 34501290 10.3390/jcm10173842 PMC8432052

[27] Bertoglio IM, Valim JML, Daffre D, Aikawa NE, Silva CA, Bonfá E et al (2021) Poor prognosis of COVID-19 acute respiratory distress syndrome in lupus erythematosus: nationwide cross-sectional population study of 252 119 patients. ACR Open Rheumatol 3(11):804–811 34423922 10.1002/acr2.11329 PMC8593786

[28] Raiker R, Pakhchanian H, DeYoung C, Gupta L, Kardeş S, Ahmed S et al (2021) Short term outcomes of COVID-19 in lupus: propensity score matched analysis from a nationwide multi-centric research network. J Autoimmun 125:102730 34649723 10.1016/j.jaut.2021.102730 PMC8501265

[29] Liew J, Gianfrancesco M, Harrison C, Izadi Z, Rush S, Lawson-Tovey S et al (2022) SARS-CoV-2 breakthrough infections among vaccinated individuals with rheumatic disease: results from the COVID-19 Global Rheumatology Alliance provider registry. RMD Open 8(1):e002187 35387864 10.1136/rmdopen-2021-002187 PMC8987210

[30] Jiang X, Sparks J, Wallace Z, Deng X, Li H, Lu N et al (2023) Risk of COVID-19 among unvaccinated and vaccinated patients with systemic lupus erythematosus: a general population study. RMD Open. 10.1136/rmdopen-2022-002839 36889799 10.1136/rmdopen-2022-002839 PMC10008206

[31] van Sleen Y, van der Geest KSM, Huckriede ALW, van Baarle D, Brouwer E (2023) Effect of DMARDs on the immunogenicity of vaccines. Nat Rev Rheumatol 19(9):560–575 37438402 10.1038/s41584-023-00992-8

[32] Conrad N, Verbeke G, Molenberghs G, Goetschalckx L, Callender T, Cambridge G et al (2022) Autoimmune diseases and cardiovascular risk: a population-based study on 19 autoimmune diseases and 12 cardiovascular diseases in 22 million individuals in the UK. Lancet 400(10354):733–743 36041475 10.1016/S0140-6736(22)01349-6

[33] Ahmed S, Gupta L, Kuwana M, Pauling JD, Day J, Ravichandran N et al (2024) Correlates of breakthrough COVID-19 in vaccinated patients with systemic sclerosis: survival analysis from a multicentre international patient-reported survey. Rheumatol Int 44(1):89–97 37668836 10.1007/s00296-023-05433-z

[34] Hoff LS, Ravichandran N, Shinjo SK, Day J, Sen P, Junior JG et al (2023) COVID-19 severity and vaccine breakthrough infections in idiopathic inflammatory myopathies, other systemic autoimmune and inflammatory diseases, and healthy controls: a multicenter cross-sectional study from the COVID-19 Vaccination in Autoimmune Diseases (COVAD) survey. Rheumatol Int 43(1):47–58 36271958 10.1007/s00296-022-05229-7 PMC9589602

[35] Cook C, Cox H, Fu X, Zhang Y, Stone JH, Choi HK et al (2021) Perceived risk and associated shielding behaviors in patients with rheumatoid arthritis during the coronavirus 2019 pandemic. ACR Open Rheumatol 3(12):834–841 34498436 10.1002/acr2.11340 PMC8653208

[36] Liu C, Lee J, Ta C, Soroush A, Rogers JR, Kim JH et al (2022) Risk factors associated with SARS-CoV-2 breakthrough infections in fully mRNA-vaccinated individuals: retrospective analysis. JMIR Public Health Surveill 8(5):e35311 35486806 10.2196/35311 PMC9132195

[37] Patel NJ, Wang X, Fu X, Kawano Y, Cook C, Vanni KMM et al (2023) Factors associated with COVID-19 breakthrough infection among vaccinated patients with rheumatic diseases: a cohort study. Semin Arthritis Rheum 58:152108 36347211 10.1016/j.semarthrit.2022.152108 PMC9605731

[38] Boekel L, Stalman EW, Wieske L, Hooijberg F, van Dam KPJ, Besten YR et al (2022) Breakthrough SARS-CoV-2 infections with the delta (B.1.617.2) variant in vaccinated patients with immune-mediated inflammatory diseases using immunosuppressants: a substudy of two prospective cohort studies. Lancet Rheumatol. 4(6):e417–e29 35527808 10.1016/S2665-9913(22)00102-3 PMC9054068

[39] Parsons C, Rubio J, Boulougoura A, Krishfield S, Kyttaris V (2023) Predictors of a weak antibody response to COVID-19 mRNA vaccine in systemic lupus erythematosus. Rheumatol Int 43(9):1621–1627 37310438 10.1007/s00296-023-05347-w

[40] Larsen ES, Nilsson AC, Möller S, Voss AB, Johansen IS (2023) Immunogenicity and risk of disease flare after a three-dose regimen with SARS-CoV-2 vaccination in patients with systemic lupus erythematosus: results from the prospective cohort study COVAC-SLE. Clin Exp Rheumatol 41(3):676–684 35894059 10.55563/clinexprheumatol/b8a6zb

[41] Ciaffi J, Vanni E, Mancarella L, Brusi V, Lisi L, Pignatti F et al (2023) Post-acute COVID-19 joint pain and new onset of rheumatic musculoskeletal diseases: a systematic review. Diagnostics (Basel) 13(11):1850 37296705 10.3390/diagnostics13111850 PMC10252492

[42] Schett G, Manger B, Simon D, Caporali R (2020) COVID-19 revisiting inflammatory pathways of arthritis. Nat Rev Rheumatol 16(8):465–470 32561873 10.1038/s41584-020-0451-z PMC7304381

[43] Veinot TC, Mitchell H, Ancker JS (2018) Good intentions are not enough: how informatics interventions can worsen inequality. J Am Med Inform Assoc 25(8):1080–1088 29788380 10.1093/jamia/ocy052 PMC7646885

[44] Uribe AG, Vilá LM, McGwin G Jr, Sanchez ML, Reveille JD, Alarcón GS (2004) The systemic lupus activity measure-revised, the mexican Systemic Lupus Erythematosus Disease Activity Index (SLEDAI), and a modified SLEDAI-2K are adequate instruments to measure disease activity in systemic lupus erythematosus. J Rheumatol 31(10):1934–1940 15468356

[45] Gordon C, Sutcliffe N, Skan J, Stoll T, Isenberg DA (2003) Definition and treatment of lupus flares measured by the BILAG index. Rheumatology (Oxford) 42(11):1372–1379 12810926 10.1093/rheumatology/keg382

