# Supplementary material for: Breakthrough SARS-CoV-2 infection in fully vaccinated patients with systemic lupus erythematosus: results from the COVID-19 Vaccination in Autoimmune Disease (COVAD) study
Source: Rheumatol Int. 2024 Aug 13;44(10):1923–33. doi: 10.1007/s00296-024-05682-6 (PMC11393143; doi:10.1007/s00296-024-05682-6)
Supplement: Supplementary file 1 — Supplementary file1 (DOCX 66 KB) [file 296_2024_5682_MOESM1_ESM.docx]

**Breakthrough SARS-CoV-2 infection in fully vaccinated patients with systemic lupus erythematosus: results from the COVID-19 Vaccination in Autoimmune Disease (COVAD) study**

**SUPPLEMENTARY MATERIAL**

**Authors**

Leonardo Palazzo^1^, Julius Lindblom^1^, Emelie Kihlgren Olsson^1^, Elena Nikiphorou^2,3^, Chris Wincup^4,5^, Sreoshy Saha^6^, Syahrul Sazliyana Shaharir^7^, Wanruchada Katchamart^8^, Phonpen Akarawatcharangura Goo^9^, Lisa Traboco^10^, Yi-Ming Chen^11,12^, James B. Lilleker^13,14,^, Arvind Nune^15^, John D Pauling^16,17^, Vishwesh Agarwal^18^, Dey Dzifa^19^, Carlos Enrique Toro Gutierrez^20^, Carlo V. Caballero-Uribe^21^, Hector Chinoy^13,22,23^, COVAD Study Group^24^, Vikas Agarwal^25#^, Latika Gupta^13,26^, Ioannis Parodis^1,27^*^#^

**Affiliations**

^1^ Division of Rheumatology, Department of Medicine Solna, Karolinska Institutet and Karolinska University Hospital, Stockholm, Sweden

^2^ Centre for Rheumatic Diseases, King’s College London, London, United Kingdom

^3^ Rheumatology Department, King’s College Hospital, London, United Kingdom

^4^ Division of Medicine, London, Department of Rheumatology, Rayne Institute, University College London, United Kingdom

^5^ Centre for Adolescent Rheumatology Versus Arthritis at UCL, UCLH, GOSH, London, United Kingdom

^6^ Mymensingh Medical College, Mymensingh, Bangladesh

^7^ Faculty of Medicine, Cheras, Universiti Kebangsaan Malaysia, Kuala Lumpur, Malaysia

^8^ Division of Rheumatology, Department of Medicine, Faculty of Medicine Siriraj Hospital, Mahidol University, Bangkok, Thailand

^9^ Department of Medicine, Queen Savang Vadhana Memorial Hospital, Chonburi, Thailand

^10^ Section of Rheumatology, Department of Medicine, St. Luke's Medical Center-Global City, Taguig, Philippines

^11^ Division of Allergy, Immunology and Rheumatology, Department of Internal Medicine, Taichung Veterans General Hospital, Taichung City, Taiwan, Republic of China

^12^ Department of Medical Research, Taichung Veterans General Hospital, Taichung, Taiwan, Republic of China

^13^ Division of Musculoskeletal and Dermatological Sciences, Centre for Musculoskeletal Research, School of Biological Sciences, Faculty of Biology, Medicine and Health, Manchester Academic Health Science Centre, The University of Manchester, Manchester, Manchester, United Kingdom

^14^ Manchester Centre for Clinical Neurosciences, Salford, Salford Royal NHS Foundation Trust, United Kingdom

^15^ Southport and Ormskirk Hospital NHS Trust, Southport, United Kingdom

^16^ Bristol Medical School Translational Health Sciences, Health Sciences, Bristol, United Kingdom

^17^ Department of Rheumatology, North Bristol NHS Trust, Bristol, United Kingdom

^18^ Mahatma Gandhi Mission Medical College, Navi Mumbai, Maharashtra, India

^19^ Rheumatology Unit, Department of Medicine and Therapeutics, University of Ghana Medical School, College of Health Sciences, Korle-Bu, Accra, Ghana

^20^ Reference Center for Osteoporosis, Rheumatology and Dermatology, Pontificia Universidad Javeriana Cali, General Director, Cali, Colombia

^21^ Department of Medicine, Hospital Universidad del Norte, Barranquilla, Atlantico, Colombia

^22^ National Institute for Health Research Manchester Biomedical Research Centre, Manchester University NHS Foundation Trust, The University of Manchester, Manchester, United Kingdom

^23^ Department of Rheumatology, Salford Royal Hospital, Northern Care Alliance NHS Foundation Trust, Salford, United Kingdom

^24^ The complete list of members of the COVAD Study Group as well as their affiliations are provided in the Supplementary Material

^25^ Department of Clinical Immunology and Rheumatology, Sanjay Gandhi Postgraduate Institute of Medical Sciences, Lucknow, India

^26^ Department of Rheumatology, Royal Wolverhampton Hospitals NHS Trust, Wolverhampton, United Kingdom

^27^ Department of Rheumatology, Faculty of Medicine and Health, Örebro University, Örebro, Sweden

*** Corresponding author**

Dr. Ioannis Parodis, MD, PhD

Division of Rheumatology, Department of Medicine Solna, Karolinska Institutet and Karolinska University Hospital, Stockholm, Sweden. ORCID ID: 0000-0002-4875-5395, Phone: +46722321322

E-mail: ioannis.parodis@ki.se

**^#^ These authors contributed equally as senior authors**

TABLE OF CONTENTS

[Supplementary Figures 6](#_Toc166321235)

[Supplementary Figure S1. Flow diagram of survey respondents included in the study. 6](#_Toc166321236)

# **Supplementary Figures**

## ****

## **Supplementary Figure S1.** Flow diagram of survey respondents included in the study.

AE: adverse event; HC: healthy controls; nrAID: non-rheumatic autoimmune disease; RA: rheumatoid arthritis; rAID: rheumatic autoimmune disease; SLE: systemic lupus erythematosus.
